# Differential Remodeling of the Macular Neovascular Network under Different Anti-VEGF Agents in Age-Related Macular Degeneration

**DOI:** 10.1016/j.xops.2026.101336

**Published:** 2026-07-28

**Authors:** Emanuele Crincoli, Enrico Borrelli, Maria Cristina Savastano, Fiammetta Catania, Matteo Mario Carlà, Daniela Bacherini, Tomaso Caporossi, Alessandra Scampoli, Federico Giannuzzi, Clara Rizzo, Veronica Vallino, Giacomo Boscia, Alfonso Savastano, Michele Reibaldi, Stanislao Rizzo

**Affiliations:** 1 Ophthalmology Department, “Fondazione Policlinico Universitario A. Gemelli, IRCCS”, Rome, Italy; 2 Ophthalmology Department, Catholic University “Sacro Cuore”, Rome, Italy; 3 Department of Surgical Sciences, University of Turin, Turin, Italy; 4 Department of Ophthalmology, “City of Health and Science” Hospital, Turin, Italy; 5 Department of Ophthalmology, Hopital Fondation Adolphe De Rothschild, Paris, France; 6 Department of Neurosciences, Psychology, Drug Research, and Child Health, Eye Clinic, University of Florence, AOU Careggi, Florence, Italy; 7 Vitreoretinal Surgery Unit, Isola Tiberina - Gemelli Isola Hospital, Rome, Italy; 8 Department of Ophthalmology, Ospedale Generale Regionale F. Miulli - Acquaviva delle Fonti (Ba), Italy; 9 Department of Ophthalmology, Libera Università Mediterranea Degennaro - Casamassima (Ba), Italy; 10 Consiglio Nazionale delle Ricerche, Istituto di Neuroscienze, Pisa, Italy

**Keywords:** Age-related macular degeneration, Anti-VEGF therapy, Faricimab, Angiopoietin-2, Macular neovascularization

## Abstract

**Purpose:**

To compare treatment-induced changes in OCT B-scan and OCT angiography (OCTA)–derived characteristics of the macular neovascular (MNV) network among different anti-VEGF agents in neovascular age-related macular degeneration (nAMD).

**Design:**

Multicenter retrospective observational cohort study.

**Subjects:**

A total of 120 eyes from 102 patients with nAMD treated with ranibizumab 0.5 mg every 4 weeks (n = 30), aflibercept 2.0 mg every 4 weeks (n = 30), aflibercept 8.0 mg every 12 weeks (n = 30), or faricimab 6.0 mg every 8 weeks (n = 30) in a treat-and-extend regimen.

**Methods:**

Eyes with good-quality OCT B-scan and OCTA acquisitions at baseline and 1-year follow-up were included. OCT B-scan variables included pigment epithelium detachment (PED) height, PED homogeneity, and presence of intraretinal cystoid lesions and hyperreflective foci. OCT angiography variables included greatest linear diameter (GLD), MNV area, vessel density (VD), vessel length density (VLD), and VD index (VDI).

**Main Outcome Measures:**

Between-group differences in OCT B-scan and OCTA parameters at 1-year follow-up, adjusted for baseline values and clinical covariates using multivariable regression with cluster-robust standard errors.

**Results:**

Baseline characteristics were comparable across groups. Significant between-group differences were observed at 1 year for GLD (*P* = 0.003), MNV area (*P* = 0.001), VD (*P* < 0.001), VLD (*P* = 0.002), and VDI (*P* < 0.001). Faricimab-treated eyes consistently showed the lowest adjusted 1-year values for all OCTA endpoints, with a mean VDI of 1.150 ± 0.07 compared with 1.453 ± 0.05 for ranibizumab, 1.334 ± 0.06 for aflibercept 2 mg, and 1.231 ± 0.06 for aflibercept 8 mg. Structural OCT outcomes differed significantly for PED height (*P* = 0.001) and PED homogeneity (*P* = 0.007), with faricimab showing the lowest PED height and highest homogeneity. No significant differences were observed for qualitative variables.

**Conclusions:**

Dual VEGF-A/angiopoietin-2 inhibition with faricimab induces greater anatomical remodeling of the MNV network compared with VEGF-A inhibition alone, with a distinct pattern characterized by preferential impact on larger-caliber vessels. These findings suggest that targeting the angiopoietin-2 pathway extends the morphological effects of anti-VEGF therapy beyond peripheral capillary pruning. Long-term studies are needed to determine whether deeper anatomical suppression translates into improved clinical outcomes or influences the trajectory of macular atrophy.

**Financial Disclosure(s):**

Proprietary or commercial disclosure may be found in the Footnotes and Disclosures at the end of this article.

Age-related macular degeneration (AMD) is the main cause of irreversible visual impairment among retinal diseases, and its prevalence is expected to increase substantially over the coming decades as a consequence of population aging.^1^ Neovascular AMD (nAMD) accounts for approximately 80% of cases of severe vision loss related to AMD.^2^ Since the introduction of anti-VEGF therapy, the visual prognosis of nAMD has significantly improved, transforming a disease once characterized by inevitable progression into a condition that can be effectively controlled in most patients.^3^ Nevertheless, despite the excellent efficacy of currently available anti-VEGF agents in suppressing retinal exudation, their ability to induce durable modifications of the underlying macular neovascular (MNV) architecture remains limited. As a result, most patients require long-term treatment. The recent clinical introduction of faricimab, a bispecific monoclonal antibody targeting both VEGF-A and angiopoietin-2 (Ang-2), has added an additional therapeutic strategy to the management of nAMD. Angiopoietin-22, acting through the Tie-2 receptor, plays a key role in vascular destabilization, inflammatory signaling, and pathological angiogenesis. Combined inhibition of the VEGF-A and Ang-2 pathways has therefore been hypothesized to exert a more pronounced effect on the growth, maturation, and survival of pathological neovascular complexes, potentially extending beyond the mere control of exudation.^4^^,^^5^ Importantly, experimental studies have suggested that Tie-2 expression appears to be preferentially associated with larger-caliber vessels, whereas Ang-2 is mainly expressed in core neovessels that are thought to drive persistence and resprouting of neovascularization.^6^^,^^7^ From a morphological perspective, inhibition of this pathway may therefore preferentially affect the main vascular trunks and central scaffold of the MNV, potentially leading to structural simplification and, in some cases, partial regression of the neovascular network.^8^

To date, the availability of faricimab in routine clinical practice has allowed these hypotheses to be explored in clinical settings. The aim of the present study is to compare treatment-induced changes in OCT B-scan and OCT angiography (OCTA)–derived characteristics of the MNV network among different anti-VEGF agents over a 1-year follow-up period, using a treat-and-extend (TE) regimen in a real-life population.

## Methods

This multicentric retrospective observational study conducted in 3 different specialized centers (Policlinico Agostino Gemelli in Rome; Molinette Hospital in Turin; Isola Tiberina - Gemelli Isola Hospital) between January 2021 and November 2025 was held in accordance with the principles of the Declaration of Helsinki and was approved by the Catholic University of the Sacred Heart Ethics Committee. Written informed consent was obtained from all included patients.

Electronic records and multimodal imaging of patients diagnosed with exudative AMD and treated with anti-VEGF intravitreal injection treatment were retrospectively collected and analyzed. The diagnosis of exudative AMD was posed in the presence of an OCTA-documented MNV in the context of retinal signs of AMD including serous or reticular drusen and pigmentary irregularities.^9^ Type I, type II, type III, and mixed MNV were included. Inclusion criteria regarding the anti-VEGF treatment were duration of treatment >12 months, no drug switch during the follow-up, and good adherence to the treatment schedule. Only patients treated with one of the following protocols were included:

1. Ranibizumab 0.5 mg every 4 weeks: TE regimen with adjustment of the interval by 2 weeks ± loading phase (3 monthly injections) in case of naïve eyes^10^ (Rq4 GROUP)
2. Aflibercept 2.0 mg every 4 weeks: TE regimen with adjustment of the interval by 2 weeks ± loading phase (3 monthly injections) in case of naïve eyes^11^ (A2q4 GROUP)
3. Aflibercept 8.0 mg every 12 weeks: TE regimen with adjustment of the interval by 4 weeks (every 8 weeks-every 16 weeks) ± loading phase (3 monthly injections) in case of naïve eyes^11^ (A8q12 GROUP)
4. Faricimab 6.0 mg every 8 weeks: TE regimen with adjustment of the interval by 4 weeks ± loading phase (4 monthly injections) in case of naïve eyes^12^ (F6q8 GROUP)

Additional inclusion criteria were availability of a good quality OCT B-scan and OCTA examination at baseline and at 1-year follow-up. Baseline was defined as a point in time 12 months prior to the last available OCTA acquisition. The exclusion criteria were the following: overlapping retinal diseases, history of ocular trauma or ocular trauma during the course of the follow-up, high myopia (>26 mm axial length or < –6.0 D in spherical equivalent), systemic treatment with vasodilating or vasoconstricting drugs, neoplastic diseases, pulmonary diseases, diabetes, previous vitreoretinal surgery or retinal laser treatment, and history of ocular or systemic inflammatory disease.

### Image Source and Image Processing

All OCT B-scan and OCTA acquisitions were performed with Spectralis HRA software version 5.4.7.0 (Heidelberg Engineering, Inc). All eyes were examined using the same device to avoid bias in quantitative OCTA parameters measurements.^13^ The minimum OCT-B scan acquisition protocol required for inclusion was a 6 × 6 mm (20 × 20°) horizontal volume scan centered of the fovea with an acquisition interval <120 μm and an automatic real-time tracking >9. The minimum OCTA acquisition protocol required for inclusion was a 6 × 6 mm (20° × 20°) horizontal volume scan with an isotropic lateral resolution of 5.7 μm/pixel (high resolution mode). This protocol potentially allows visualization of the smallest capillaries, whose estimated diameter is around 8 μm.^14^ For MNV related variables, a customized 40-μm slab enface segmentation at the level of the avascular zone was performed by 2 different experienced graders to allow the best possible visibility of the whole MNV network. The resulting enface section was binarized and spurious elements were removed with a denoising filter using ImageJ software (National Institutes of Health). For choriocapillaris segmentation, a slab starting from the outer boundary of the Bruch membrane (BM) and characterized by a thickness of 20 μm was selected. This thickness was chosen to account for both the mean diameter of choriocapillaris vessels (6-10 μm) and the convolutional effect of OCT resolution which artificially fattens biological tissues. The outer boundary of the BM was identified as a line passing ∼4 μm below the inner boundary of the BM based on mean BM thickness.^15^^,^^16^ A scanning quality (Q index) >15 was required for validation of the acquisition.

### Study Outcomes and Considered Variables

The main outcome of the study was to compare changes in MNV OCT B-scan and OCTA characteristics after 1 year of treatment between each group. The primary endpoint was defined as the comparison of OCT B-scan and OCTA parameters at 1-year follow-up between treatment groups, adjusted for baseline values and clinical confounders.

The following OCT B-scan variables were considered:

1. Mean pigment epithelium detachment (PED) height: it was defined as the vertical linear distance from the hyperreflective line of BM to the inner margin of the hyperreflective line of the retinal pigment epithelium (RPE) layer.^17^ The mean of the maximal height from all sections including the PED was considered.
2. Pigment epithelium detachment homogeneity: it was defined as the percentage of pixels showing a luminance within 1 standard deviation of the mean luminance of the pixels contained within the PED region. The mean PED homogeneity of all scans including the PED was considered.
3. Presence of intraretinal cystoid lesions (ICLs): the presence or absence of ICLs was qualitatively assessed as a binomial variable (presence vs. absence) by 2 experienced graders. Evaluation was performed assessing the presence of ICLs only in cross-sectional slabs including the PED.
4. Presence of hyperreflective foci (HFs) in the retina: HFs were defined as well-circumscribed discrete lesions with a diameter >20 μm and with equal or greater reflectivity than that of the RPE and were detected in ≥1 available OCT scan. Hyperreflective foci were defined as outer retina HFs when located between the outer border of the RPE and the inner border of the outer nuclear layer. Hyperreflective foci were considered as inner retina HFs when located between the inner margin of the outer nuclear layer and the internal limiting membrane. Their presence or absence was qualitatively assessed as a binomial variable (presence or absence) by 2 experienced graders.

As concerns OCTA, the following variables were considered:

1. Greatest linear diameter (GLD): it was defined as the longest linear distance between 2 opposite margins of the MNV.
2. Macular neovascular area: the customized enface OCTA slab was segmented to contour the MNV profile, converted to gray scale and binarized using an automatic mean-based thresholding as previously described.^18^ All processing procedures were performed using ImageJ software. Pixel to mm conversion was performed using Spectralis caliper as a reference.
3. Macular neovascular vessel density (VD): defined as the percentage of the area of the binarized segmented MNV covered by white (vascular) pixels. Macular neovascular VD calculation was performed on segmented and binarized MNV image using Vessel Analysis plug in in ImageJ.
4. Macular neovascular vessel length density (VLD): defined as the percentage of the area of the binarized segmented and skeletonized MNV covered by white (vascular) pixels. Calculation of VLD was performed using Vessel analysis plug in on ImageJ (ImageJ software).
5. Macular neovascular VD index (VDI): was defined as the ratio between VD and VLD of the MNV in each point in time.^19^ This parameter aims to describe the density of large caliber vessels within the vascular network. In fact, a high VD/VLD ratio is characteristic of a network in which large caliber vessels are well represented. A decrease in this ratio during the follow-up would therefore mean a prevalent regression of large vessel trunks within the MNV.

### Statistical Analysis

Statistical analysis was performed using Python (version 3.10; Python Software Foundation), with the use of standard scientific libraries for statistical modeling and data analysis.

Sample size calculation was performed considering an acceptable alpha error of 5%, a beta error of 20% and a clinically significant reduction in VD of 5%. Normality of the distribution for quantitative variables was assessed graphically and confirmed with Shapiro–Wilk test. Continuous variables are reported as mean ± standard deviation, and categorical variables as counts and percentages. Baseline demographic and imaging characteristics were compared across treatment groups using 1-way analysis of variance for continuous variables and χ^2^ test or Fisher exact test, as appropriate, for categorical variables. These analyses were considered descriptive and aimed at assessing baseline comparability between groups.

The primary analysis evaluated between-group differences in OCT B-scan and OCTA outcomes at 1-year follow-up using multivariable regression models, with the 1-year value of each outcome as the dependent variable and treatment group as the main independent variable. All models were adjusted for the corresponding baseline value of the outcome and for clinically relevant covariates, including age, sex, treatment-naïve status, pseudophakic status, and MNV type. To account for the inclusion of both eyes in a subset of patients, robust standard errors clustered at the patient level were used. Intergrader agreement for qualitative variables was assessed using Cohen kappa coefficient, while agreement for quantitative measurements was evaluated using the intraclass correlation coefficient. A 2-sided *P* value <0.05 was considered statistically significant.

## Results

A total of 120 eyes from 102 patients were included (30 eyes per treatment group). Both eyes were included in 18 patients (17.6%), while the remaining patients contributed a single eye. Baseline demographic and clinical characteristics were comparable across groups (Table 1). Overall mean age was 69.8 ± 7.6 years, 55.9% of eyes were from male patients, 73.5% had a history of hypertension, 53.9% were pseudophakic, and 26.5% were treatment-naïve. The mean number of intravitreal injections over the 1-year follow-up was 10.3 ± 1.9 in the ranibizumab group, 9.7 ± 1.8 in the aflibercept 2 mg group, 6.8 ± 1.4 in the aflibercept 8 mg group, and 6.5 ± 1.6 in the faricimab group. The distribution of MNV subtypes was similar among groups, with type I lesions being predominant.

**Table 1:** Baseline Demographic and Clinical Characteristics by Treatment Group

Characteristic	Rq4	A2q4	A8q12	F6q8	*P*
Age (yr)	68.7 ± 8.7	70.9 ± 8.0	69.2 ± 7.3	70.8 ± 6.7	0.678
Male	17/25 (68.0%)	13/25 (52.0%)	17/27 (63.0%)	10/25 (40.0%)	0.191
HTN	20/25 (80.0%)	17/25 (68.0%)	18/27 (66.7%)	20/25 (80.0%)	0.548
Pseudophakic	15/25 (60.0%)	15/25 (60.0%)	12/27 (44.4%)	13/25 (60.0%)	0.624
Treatment-naive	9/25 (36.0%)	6/25 (24.0%)	6/27 (22.2%)	6/25 (24.0%)	0.665
MNV type (I/II/III/mixed)	17/2/2/4	13/7/1/4	11/5/5/6	11/7/1/6	0.318

A2q4 = aflibercept 2.0 mg every 4 weeks; A8q12 = aflibercept 8.0 mg every 12 weeks; F6q8 = faricimab 6.0 mg every 8 weeks; HTN = arterial hypertension; MNV = macular neovascularization; Rq4 = ranibizumab 0.5 mg every 4 weeks.

Baseline demographic and clinical characteristics of the study population stratified by treatment regimen.

**Figure 1:**
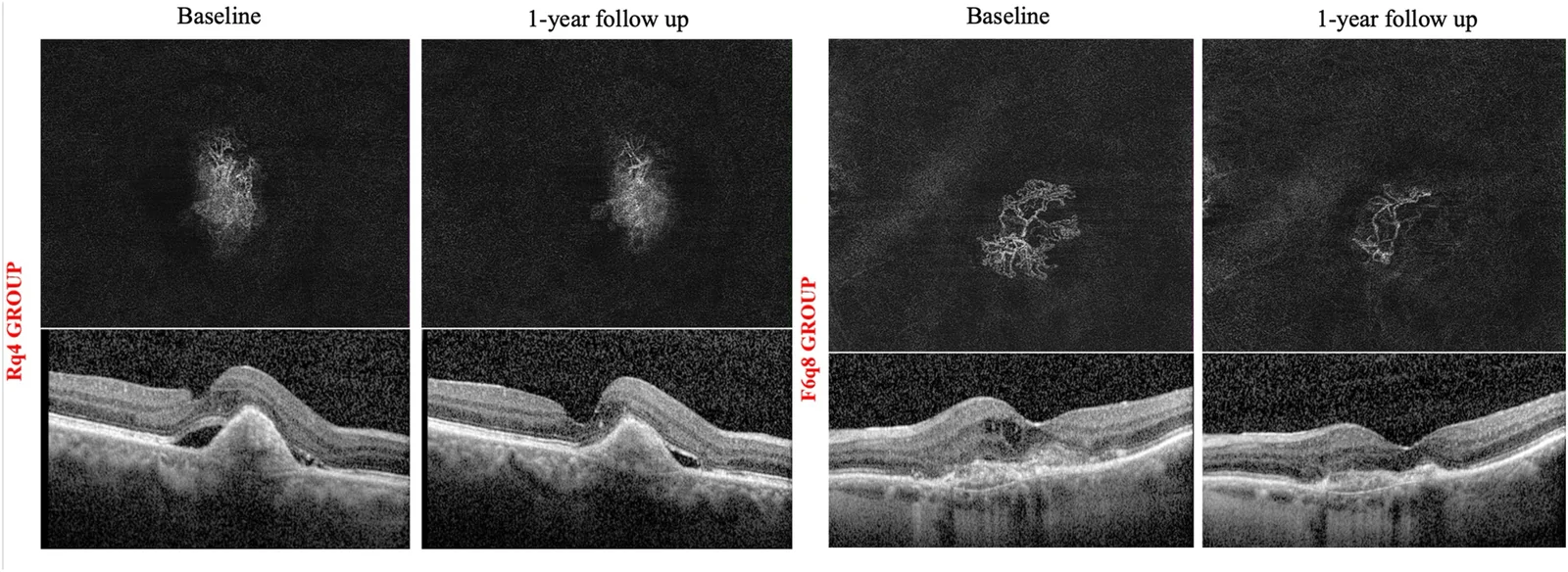
Representative OCTA and OCT B-scan changes over 1-year follow-up in different treatment groups. Representative examples of en face OCTA images of macular neovascularization (upper panels) and corresponding OCT B-scan sections (lower panels) at baseline and after 1 year of treatment. The left panels show an eye treated with a ranibizumab Q4 treat-and-extend regimen (Rq4), while the right panels show an eye treated with a faricimab Q8 treat-and-extend regimen (F6q8). Compared with baseline, the faricimab-treated eye shows a marked reduction in neovascular network extent and simplification of vascular architecture on OCTA, together with flattening and increased homogeneity of the pigment epithelium detachment on structural OCT. F6q8 = faricimab 6.0 mg every 8 weeks; OCTA = OCT angiography; Rq4 = ranibizumab 0.5 mg every 4 weeks.

**Figure 2:**
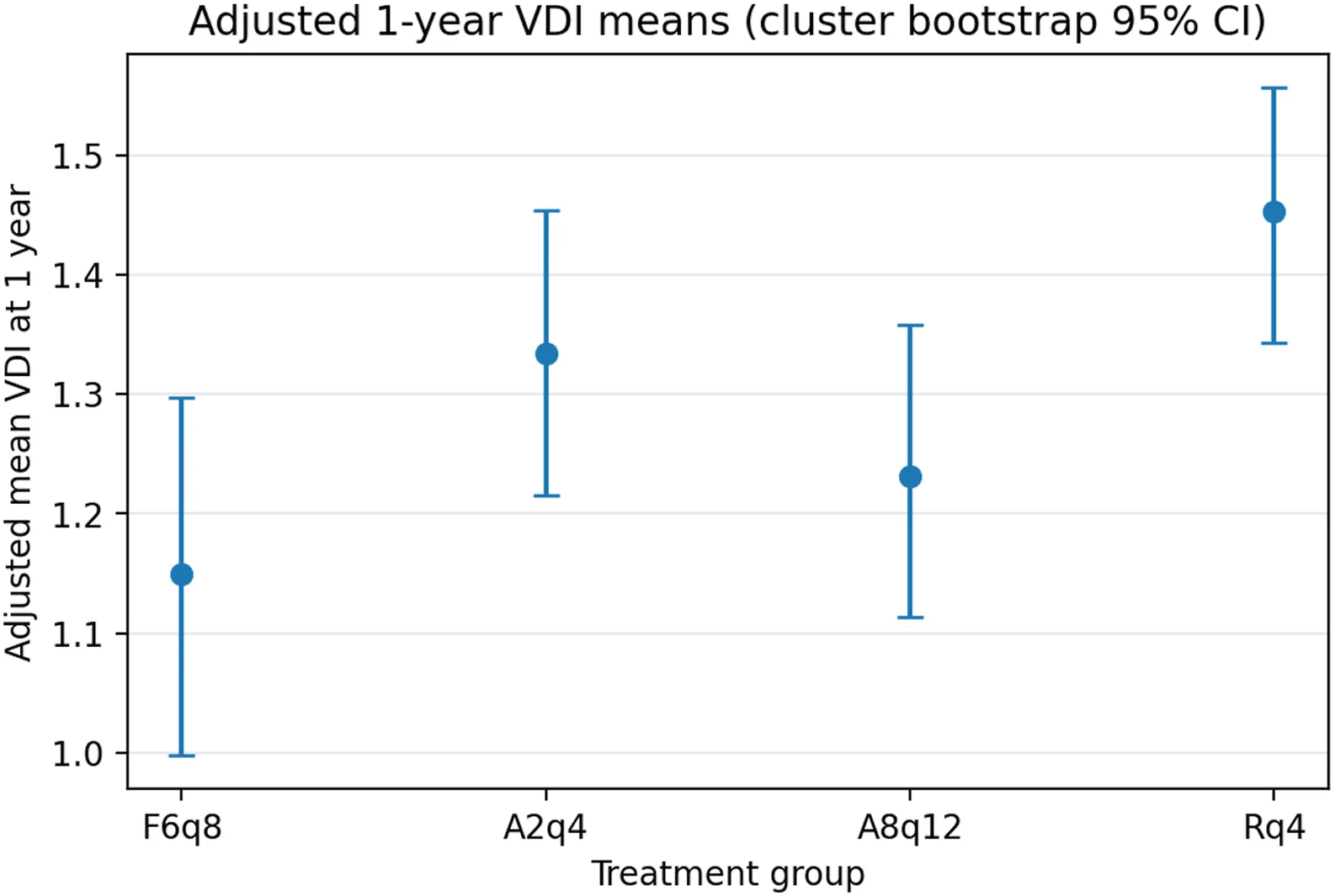
Adjusted 1-year vessel diameter index by treatment group. Adjusted mean VDI values at 1-year follow-up by treatment group, estimated from multivariable linear regression models. Points represent adjusted means and error bars indicate cluster-bootstrap 95% CIs, accounting for within-patient correlation. Models were adjusted for baseline VDI and relevant clinical covariates. A2q4 = aflibercept 2.0 mg every 4 weeks; A8q12 = aflibercept 8.0 mg every 12 weeks; CI = confidence interval; F6q8 = faricimab 6.0 mg every 8 weeks; Rq4 = ranibizumab 0.5 mg every 4 weeks; VDI = vessel density index.

**Figure 3:**
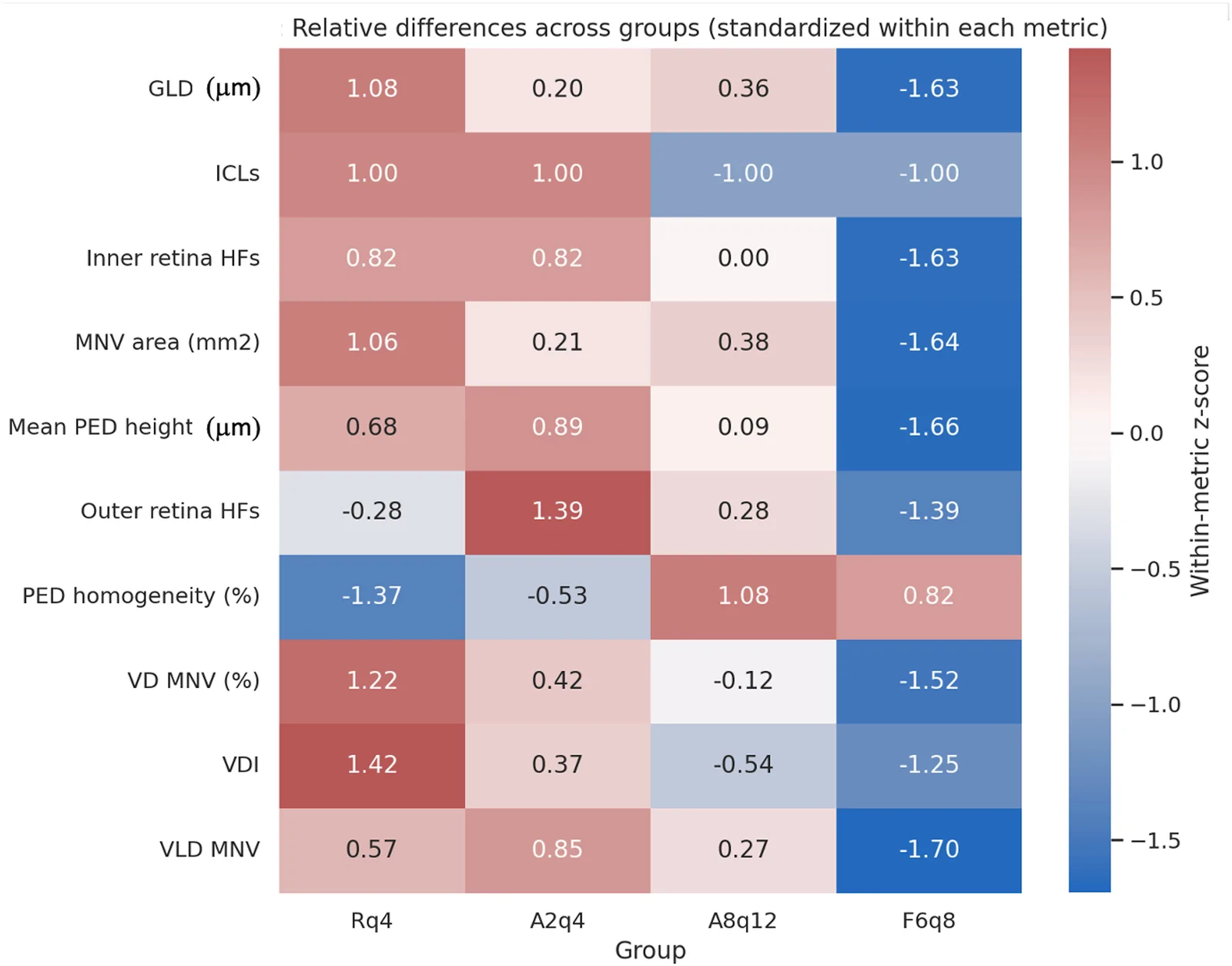
Relative differences across treatment groups for OCT B-scan and OCTA metrics at 1 year. Heatmap showing relative differences across treatment groups for OCT B-scan and OCTA metrics at 1-year follow-up. For each metric, values were standardized within the metric (z-scores) to allow comparison of relative patterns across groups. Positive values (red) indicate higher relative values, whereas negative values (blue) indicate lower relative values within each metric. This visualization highlights distinct group-specific profiles across structural and vascular parameters. A2q4 = aflibercept 2.0 mg every 4 weeks; A8q12 = aflibercept 8.0 mg every 12 weeks; F6q8 = faricimab 6.0 mg every 8 weeks; GLD = greatest linear diameter; HF = hyperreflective foci; ICL = intraretinal cystoid lesion; MNV = macular neovascularization; PED = pigment epithelium detachment; OCTA = OCT angiography; Rq4 = ranibizumab 0.5 mg every 4 weeks; VD = vessel density; VDI = vessel density index; VLD = vessel length density.

Baseline demographic, clinical, OCT B-scan, and OCTA characteristics were comparable across groups (Tables 1 and 2). Between-group differences at 1-year follow-up were assessed using multivariable regression models adjusted for the corresponding baseline value and clinically relevant covariates (Table 3). Significant overall between-group differences were observed at 1 year for the primary OCTA outcomes, including GLD (*P* = 0.003), MNV area (*P* = 0.001), VD (*P* < 0.001), VLD (*P* = 0.002), and VDI (*P* < 0.001) (see Fig 1 and 2). Across these endpoints, the faricimab group consistently showed the lowest adjusted 1-year values, indicating a greater reduction in lesion size and a more pronounced rarefaction of the neovascular network on OCTA (see Fig 3). Structural OCT outcomes also differed across groups at 1 year, with significant overall between-group effects for PED height (*P* = 0.001) and PED homogeneity (*P* = 0.007); again, faricimab-treated eyes showed the lowest adjusted PED height and the highest adjusted PED homogeneity at follow-up.

**Table 2:** Baseline OCT B-Scan and OCT Angiography Parameters by Treatment Group

Baseline	Rq4	A2q4	A8q12	F6q8	*P*
GLD (μm)	1627.3 ± 657.2	1863.6 ± 514.8	2062.4 ± 498.4	1879.5 ± 768.0	0.098
MNV area (mm^2^)	2.87 ± 0.82	2.87 ± 0.71	2.94 ± 0.87	2.74 ± 0.79	0.841
VD MNV (%)	39.43 ± 5.53	39.40 ± 5.25	39.62 ± 3.96	38.40 ± 4.26	0.799
VLD MNV	29.41 ± 4.55	27.37 ± 5.12	30.11 ± 4.94	27.89 ± 6.22	0.208
VDI	1.48 ± 0.57	1.33 ± 0.46	1.31 ± 0.51	1.27 ± 0.44	0.448
Mean PED height (μm)	302.564 ± 34.74	290.366 ± 30.99	294.379 ± 40.66	286.218 ± 36.76	0.427
PED homogeneity (%)	69.74 ± 7.56	71.45 ± 8.63	69.07 ± 7.83	65.58 ± 9.97	0.107
ICLs	9/25 (36.0%)	9/25 (36.0%)	13/27 (48.1%)	11/25 (44.0%)	0.758
Inner retina HFs	11/25 (44.0%)	13/25 (52.0%)	14/27 (51.8%)	14/25 (56.0%)	0.233
Outer retina HFs	16/25 (64.0%)	19/25 (76.0%)	22/27 (81.5%)	15/25 (60.0%)	0.288

A2q4 = aflibercept 2.0 mg every 4 weeks; A8q12 = aflibercept 8.0 mg every 12 weeks; F6q8 = faricimab 6.0 mg every 8 weeks; GLD = greatest linear diameter; HF = hyperreflective foci; ICL = intraretinal cystoid lesion; MNV = macular neovascularization; PED = pigment epithelium detachment; Rq4 = ranibizumab 0.5 mg every 4 weeks; VD = vessel density; VDI = vessel density index; VLD = vessel length density.

Baseline OCT B-scan and OCT angiography characteristics stratified by treatment group. Data are reported as mean ± standard deviation for continuous variables and as number (percentage) for categorical variables. These analyses are descriptive and intended to assess baseline comparability across groups.

**Table 3:** Adjusted OCT B-Scan and OCT Angiography Outcomes at 1-Yr Follow-Up by Treatment Group

1-Yr Follow-up	Rq4	A2q4	A8q12	F6q8	*P*
GLD (μm)	1531.54 ± 70.25	1455.15 ± 76.77	1469.38 ± 74.28	1296.50 ± 89.65	0.003
MNV area (mm^2^)	1.816 ± 0.133	1.718 ± 0.126	1.737 ± 0.107	1.503 ± 0.159	0.001
VD MNV (%)	36.04 ± 0.75	32.82 ± 0.66	30.61 ± 0.64	24.92 ± 1.17	<0.001
VLD MNV	24.83 ± 1.19	25.40 ± 1.17	24.24 ± 1.02	20.30 ± 1.77	0.002
VDI	1.453 ± 0.05	1.334 ± 0.06	1.231 ± 0.06	1.150 ± 0.07	<0.001
Mean PED height (μm)	269.07 ± 34.04	271.29 ± 37.59	262.70 ± 39.45	243.98 ± 38.38	0.001
PED homogeneity (%)	76.89 ± 1.18	79.34 ± 1.17	84.09 ± 1.09	83.31 ± 1.23	0.007
ICLs	4/25 (16.0%)	4/25 (16.0%)	2/27 (7.4%)	2/25 (8.0%)	0.083
Inner retina HFs	8/25 (32.0%)	8/25 (32.0%)	7/27 (25.9%)	5/25 (20.0%)	0.067
Outer retina HFs	10/25 (40.0%)	13/25 (52.0%)	11/27 (40.7%)	8/25 (32.0%)	0.091

A2q4 = aflibercept 2.0 mg every 4 weeks; A8q12 = aflibercept 8.0 mg every 12 weeks; F6q8 = faricimab 6.0 mg every 8 weeks; GLD = greatest linear diameter; HF = hyperreflective foci; ICL = intraretinal cystoid lesion; MNV = macular neovascularization; PED = pigment epithelium detachment; Rq4 = ranibizumab 0.5 mg every 4 weeks; VD = vessel density; VDI = vessel density index; VLD = vessel length density.

Adjusted OCT B-scan and OCT angiography outcomes at 1-yr follow-up derived from multivariable regression models. Values are reported as adjusted means ± standard error. Between-group comparisons (*P*∗) reflect global treatment effects at 1 yr after adjustment for the corresponding baseline value of each outcome, age, sex, treatment-naïve status, pseudophakia, and MNV type, with robust standard errors clustered at the patient level.

The prevalence of ICLs and HF decreased over time in all groups; adjusted logistic regression analyses for qualitative outcomes showed no statistically significant between-group differences at 1 year, although complete resolution of ICLs occurred more frequently in faricimab-treated eyes. Similarly, no significant differences were observed for inner retinal HF or outer retinal HF. In 5 patients in whom both eyes were included and treated with different anti-VEGF agents, the eye treated with faricimab consistently showed a greater 1-year reduction in GLD and a more pronounced decrease in VDI compared with the fellow eye treated with another molecule; these observations were considered qualitative and not intended for statistical inference.

## Discussion

Intravitreal anti-VEGF therapy has profoundly modified the natural history of nAMD, allowing durable control of exudation and functional stabilization in the majority of patients. Nevertheless, from a structural standpoint, MNVs often persist despite long-term treatment, showing that the suppression of vascular permeability is not necessarily associated to elimination or profound remodeling of the underlying neovascular complex.

The introduction of OCTA has provided the opportunity to observe in vivo the evolution of neovascular networks under anti-VEGF pressure, revealing that “regression” is not a univocal phenomenon. Early qualitative OCTA observations first highlighted that anti-VEGF therapy commonly induces pruning of peripheral capillaries and simplification of the neovascular network without complete disappearance of flow, suggesting partial remodeling rather than true vascular involution. Spaide et al^20^ described this phenomenon as a process of vascular “abnormalization,” in which neovascular complexes become progressively less dense and less complex while maintaining a persistent central scaffold. Quantitative OCTA analyses later confirmed these observations: Montesel et al^21^ reported a reduction in MNV area and VD of approximately 20% and 12%, respectively, after the loading phase with aflibercept, supporting the concept that VEGF-A inhibition alone can induce measurable structural remodeling of the neovascular network.

Subsequent longitudinal OCTA studies confirmed that reductions in lesion area, VD, and branching complexity are common findings during treatment, while larger-caliber vascular trunks often persist over time, supporting the concept that VEGF-A inhibition alone preferentially affects the more immature and VEGF-dependent components of the network.

Importantly, several studies have emphasized that apparent “regression” may correspond to qualitative transformations of the lesion rather than to its eradication. In type 2 MNV, Dolz-Marco et al^22^ demonstrated that anti-VEGF therapy can induce a progressive relocation of neovascular tissue beneath the RPE, resulting in a transition toward a type 1 configuration. Similarly, in type 3 MNV, regression has been shown to preferentially involve the intraretinal anastomotic stalk, while choroidal components may persist, further highlighting the subtype-specific and spatially heterogeneous response of neovascular tissue to therapy. These findings highlight that regression may reflect phenotypic reorganization and compartmentalization of neovascular tissue rather than loss of vascular elements.

Long-term OCTA analyses further reinforce this view. Levine et al showed that neovascular complexes frequently remain detectable over extended follow-up, despite marked changes in morphology, density, and apparent activity. The persistence of a residual vascular scaffold suggests that anti-VEGF therapy may stabilize rather than eliminate neovascular tissue, raising questions about the biological meaning of progressive rarefaction and its long-term consequences. In parallel, more refined analyses of treated type 1 MNV have demonstrated that the spatial relationship between neovascular tissue and the surrounding choriocapillaris is not neutral. Corvi et al^23^ showed that a reduced “neo-choriocapillaris” coverage overlying type 1 MNV is associated with a higher risk of macular atrophy, suggesting that some neovascular configurations may exert a compensatory or protective role, while others may predispose to atrophic evolution.

Within this conceptual framework, the results of the present study suggest that dual VEGF-A and Ang-2 inhibition may induce a qualitatively different pattern of vascular remodeling. While all anti-VEGF agents analyzed were associated with significant reductions in MNV area, VD, and VLD, the magnitude and distribution of these changes differed across treatment strategies. Eyes treated with faricimab showed not only greater reductions in lesion size and vascular density, but also a disproportionate decrease in VDI, indicating preferential involvement of larger-caliber vascular components.

This finding is biologically plausible in light of the Ang-2/Tie-2 pathway, which plays a central role in vascular destabilization and is more prominently expressed in mature and larger vessels within pathological neovascular networks. Inhibition of this pathway, combined with VEGF-A blockade, may therefore extend the antiangiogenic effect beyond capillary pruning, promoting destabilization of the central vascular scaffold. From a morphological perspective, this pattern differs from the remodeling typically observed with VEGF-A inhibition alone and suggests a deeper structural impact on the neovascular architecture. It should be acknowledged that VDI represents an indirect composite metric derived from the ratio between VD and VLD, and therefore does not provide a direct measurement of individual vessel caliber. However, within the context of OCTA-based analyses, it has been consistently used as a surrogate marker of the relative contribution of larger-caliber vessels within the neovascular network. Accordingly, the observed reduction in VDI should be interpreted as indicative of a shift in vascular architecture toward a relative depletion of larger vascular trunks, rather than as a direct quantification of vessel diameter changes.

However, deeper anatomical suppression of neovascular tissue raises important interpretative and clinical questions. Channa et al^24^ reported that regression of choroidal neovascularization under anti-VEGF therapy can be followed by the development of macular atrophy, highlighting that elimination or involution of neovascular tissue may come at the cost of reduced trophic support to the outer retina in selected cases. These observations caution against interpreting vascular regression as an unequivocal therapeutic goal and emphasize the need to distinguish beneficial remodeling from potentially deleterious vascular loss.

Complete disappearance of the OCTA flow signal represents a further and more controversial endpoint. Savastano et al^25^ reported cases of near-complete loss of detectable neovascular flow after faricimab treatment, raising the possibility that dual VEGF-A/Ang-2 inhibition may induce deeper structural effects than VEGF-A blockade alone. However, earlier OCTA studies using other anti-VEGF molecules documented disappearance of neovascular flow that was often transient, with subsequent reappearance on follow-up imaging.^26^ In the present study, complete disappearance of the detectable OCTA flow signal was observed in a limited number of faricimab-treated eyes. While this finding is consistent with the possibility of profound structural involution, it should be interpreted with caution. Loss of detectable flow may reflect true vascular regression but may also result from residual flow below the detection threshold, segmentation limitations, or device-related constraints, particularly when using spectral-domain OCTA.

In 5 patients with both eyes included and treated with different agents (one eye receiving faricimab), the faricimab-treated eye showed greater 1-year GLD reduction and a more marked VDI decrease than the fellow eye treated with a different molecule. Although anecdotal and not suitable for formal statistical inference, these intraindividual observations reduce the likelihood that the observed effects are driven solely by population differences and suggest a molecule-specific anatomical response.

This study is limited by its retrospective design and sample size, which restrict causal inference. OCT angiography acquisitions were performed using spectral-domain technology, which may underestimate slow or deep flow and complicate interpretation of complete flow disappearance. Definitions of “regression” based on OCTA are inherently influenced by device sensitivity and segmentation accuracy. The 1-year follow-up may be insufficient to assess long-term durability of structural changes and their relationship with macular atrophy.

Taken together, these findings indicate that anti-VEGF therapy encompasses a spectrum of structural effects ranging from partial remodeling to more profound alterations of neovascular architecture. Dual VEGF-A/Ang-2 inhibition appears to shift this balance toward deeper modification of the neovascular scaffold, with potential implications for durability, retreatment burden, and long-term retinal integrity. Future studies with larger cohorts, longer follow-up, and high-resolution swept-source OCTA will be essential to clarify whether stronger anatomical suppression translates into improved long-term outcomes or modifies the trajectory of macular atrophy.
